# The Inhibitory Effects of Purple Sweet Potato Color on Hepatic Inflammation Is Associated with Restoration of NAD^+^ Levels and Attenuation of NLRP3 Inflammasome Activation in High-Fat-Diet-Treated Mice

**DOI:** 10.3390/molecules22081315

**Published:** 2017-08-08

**Authors:** Xin Wang, Zi-Feng Zhang, Gui-Hong Zheng, Ai-Min Wang, Chun-Hui Sun, Su-Ping Qin, Juan Zhuang, Jun Lu, Dai-Fu Ma, Yuan-Lin Zheng

**Affiliations:** 1 Key Laboratory for Biotechnology on Medicinal Plants of Jiangsu Province, School of Life Science, Jiangsu Normal University, Xuzhou 221116, Jiangsu Province, China; xznkywx@163.com (X.W.); 6020030110@jsnu.edu.cn (G.-H.Z.); aiminwang@jsnu.edu.cn (A.-M.W.); 6020110036@jsnu.edu.cn (C.-H.S.); qinsuping1234@163.com (S.-P.Q.); dajiangsky@163.com (J.Z.); lu-jun75@163.com (J.L.); 2 Key Laboratory of Biology and Genetic Improvement of Sweetpotato, Ministry of Agriculture, Jiangsu Xuzhou Sweetpotato Research Center, Xuzhou 221131, Jiangsu Province, China; daifuma@163.com

**Keywords:** purple sweet potato color, hepatic inflammation, NAD^+^, NLRP3 inflammasome, high-fat diet

## Abstract

Purple sweet potato color (PSPC), a class of naturally occurring anthocyanins, exhibits beneficial effects on metabolic syndrome. Sustained inflammation plays a crucial role in the pathogenesis of metabolic syndrome. Here we explored the effects of PSPC on high-fat diet (HFD)-induced hepatic inflammation and the mechanisms underlying these effects. Mice were divided into four groups: Control group, HFD group, HFD + PSPC group, and PSPC group. PSPC was administered by daily oral gavage at doses of 700 mg/kg/day for 20 weeks. Nicotinamide riboside (NR) was used to increase NAD^+^ levels. Our results showed that PSPC effectively ameliorated obesity and liver injuries in HFD-fed mice. Moreover, PSPC notably blocked hepatic oxidative stress in HFD-treated mice. Furthermore, PSPC dramatically restored NAD^+^ level to abate endoplasmic reticulum stress (ER stress) in HFD-treated mouse livers, which was confirmed by NR treatment. Consequently, PSPC remarkably suppressed the nuclear factor-κB (NF-κB) p65 nuclear translocation and nucleotide oligomerization domain protein1/2 (NOD1/2) signaling in HFD-treated mouse livers. Thereby, PSPC markedly diminished the NLR family, pyrin domain containing 3 (NLRP3) inflammasome activation, ultimately lowering the expressions of inflammation-related genes in HFD-treated mouse livers. In summary, PSPC protected against HFD-induced hepatic inflammation by boosting NAD^+^ level to inhibit NLRP3 inflammasome activation.

## 1. Introduction

Substantial evidence has highlighted the crucial role of sustained inflammation in the pathogenesis of Type 2 diabetes (T2D) during the past decades. It is well established that liver inflammation caused by high fat diet (HFD) and obesity impairs hepatic glucose and lipid metabolism, leading to local and systemic insulin resistance, ultimately contributing to the development of T2D [1,2]. In particular, accumulating evidence suggests that inhibition of hepatic inflammatory response improves HFD and obesity-induced metabolic disorders, such as insulin resistance and hyperglycemia [3,4]. Though the role of liver inflammation in the development of T2D has been widely investigated, the triggering mechanisms underlying HFD and obesity-induced hepatic inflammation remain to be elucidated.

Nicotinamide adenine dinucleotide (NAD^+^), in addition to acting as a coenzyme, has been demonstrated to act as a key mediator in regulating a wide spectrum of physiological and pathological processes, such as metabolism, circadian rhythm, aging and cancer. Accumulating evidence has indicated that the level of NAD^+^ is declined due to the compromised NAD^+^ biosynthesis and the enhanced NAD^+^ breakdown in various tissues, including liver and fat, under HFD and obese conditions [5,6]. Recently, it is well established that loss of NAD^+^ is involved in the development of HFD and obesity-induced metabolic disorders, such as steatosis, dyslipidemia and insulin resistance [7,8]. Therefore, improving NAD^+^ metabolism has emerged as a potential therapeutic strategy for T2D and its complications. However, whether the dysregulation of NAD^+^ metabolism contributes to liver inflammation during T2D and the mechanisms underlying these effects remain to be investigated.

The NLR family, pyrin domain-containing 3 (NLRP3) inflammasome is an important member of the innate immune system, which is a cytoplasmic multi-protein complex composed of NLRP3, apoptosis-associated speck-like protein containing a CARD domain (ASC) and pro-cysteine aspartate-specific protease-1 (pro-caspase-1). Over the recent past, the importance of NLRP3 inflammasome-mediated caspase-1 activation and subsequent pro-inflammatory cytokines release as one of the contributing mechanisms to T2D has been recognized [9,10]. Indeed, emerging evidence demonstrates that NLRP3 inflammasome activation leads to hepatic metaflammation, contributing to abnormal lipid and glucose metabolism, exacerbating T2D and its complications [11,12]. Nevertheless, the mechanisms of NLRP3 inflammasome activation mediated by HFD and obesity are not fully understood.

Purple sweet potato color (PSPC) is a class of naturally occurring anthocyanins that is derived from purple sweet potato storage roots that reportedly has a variety of pharmacological properties including strong antioxidant, anti-inflammatory and neuroprotective effects [13,14,15]. Our previous work revealed that PSPC treatment protected mouse liver from D-galactose-induced oxidative stress, inflammation and hepatocyte apoptosis [16,17]. Recently, our studies indicated that PSPC treatment inhibited HFD-induced hepatic insulin resistance partly via repressing oxidative stress and endoplasmic reticulum stress (ER stress) [18]. Thus, PSPC could be regarded as a suitable candidate for pharmacological intervention of T2D. However, whether PSPC attenuates HFD-induced hepatic inflammation has never been investigated.

In the present study, we hypothesized that PSPC treatment might inhibit NLRP3 inflammasome activation-mediated hepatic inflammation via improving NAD^+^ metabolism in HFD-fed mice. The improvement of NAD^+^ metabolism could be a novel protective mechanism of PSPC against T2D and its complications. This study is designed to address these issues.

## 2. Results

### 2.1. PSPC Ameliorates Obesity and Liver Injury in HFD-Treated Mice

**Figure 1:**
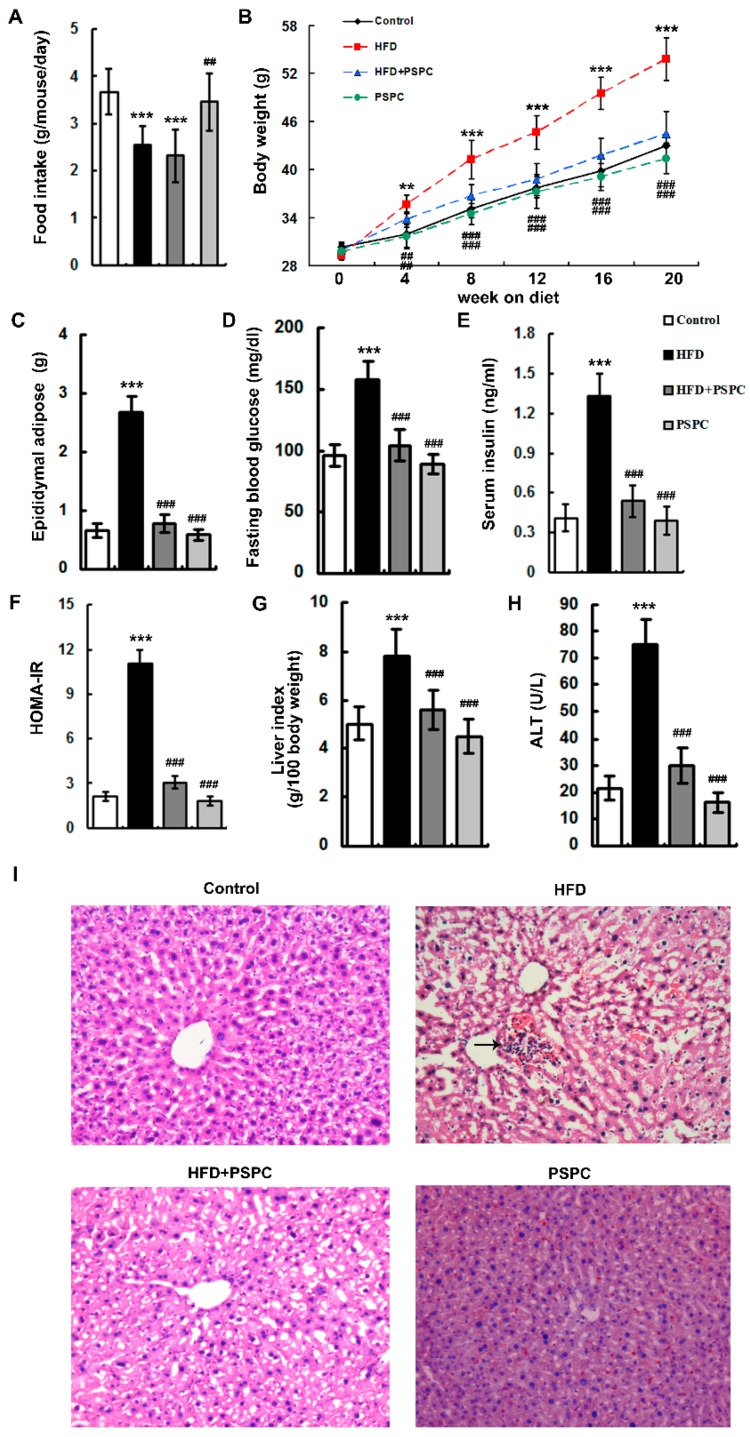
PSPC ameliorates obesity and liver injury in HFD-fed mice (*n* = 5). (**A**) Food intake in all treated groups; (**B**) Total body weight in all treated groups; (**C**) The levels of epididymal adipose tissue masses in all treated groups; (**D**) The levels of fasting blood glucose in all treated groups; (**E**) The levels of serum insulin in all treated groups; (**F**) The levels of HOMA index in all treated groups; (**G**) The levels of liver index in all treated groups; (**H**) Serum ALT activities in all treated groups; (**I**) H&E staining of liver sections in all treated groups, 200× magnification. Inflammatory cells are indicated by black arrow. All of the values are expressed as the mean ± SD. ** *p* < 0.01, *** *p* < 0.001 vs. the control group; ## *p* < 0.01, ### *p* < 0.001 vs. the HFD group.

The mice fed an ND showed higher food intake than that of mice fed HFD due to the low energy density of the ND (Figure 1A). PSPC treatment did not significantly affect the food intake in either ND-fed mice or HFD-fed mice (Figure 1A).

Feeding HFD to mice for 20 weeks induced a marked increase in body weight, as well as in epididymal adipose tissue mass (Figure 1B,C). Moreover, HFD significantly elevated the levels of fasting blood glucose, serum insulin and HOMA index in mice (Figure 1D–F).

HFD-fed mice had remarkably higher levels of liver index and serum aminotransferase (ALT) than control mice (Figure 1G,H). The results of histological analysis showed that HFD treatment caused hepatocyte hypertrophy and vacuolization and inflammatory cell infiltration in mouse livers (Figure 1I), which confirmed an occurrence of obesity-related liver injury.

Interestingly, PSPC dramatically lowered body weight, epididymal adipose tissue mass, fasting blood glucose, serum insulin, HOMA index, liver index and serum ALT, and effectively improved these histopathological changes of livers in HFD-treated mice (Figure 1). There were no significant differences in obesity and liver injuries among the HFD + PSPC, PSPC and the control groups. These results indicate that PSPC effectively ameliorates obesity and liver injuries in HFD-treated mice.

### 2.2. PSPC Attenuates Oxidative Stress in HFD-Treated Mouse Livers

**Figure 2:**
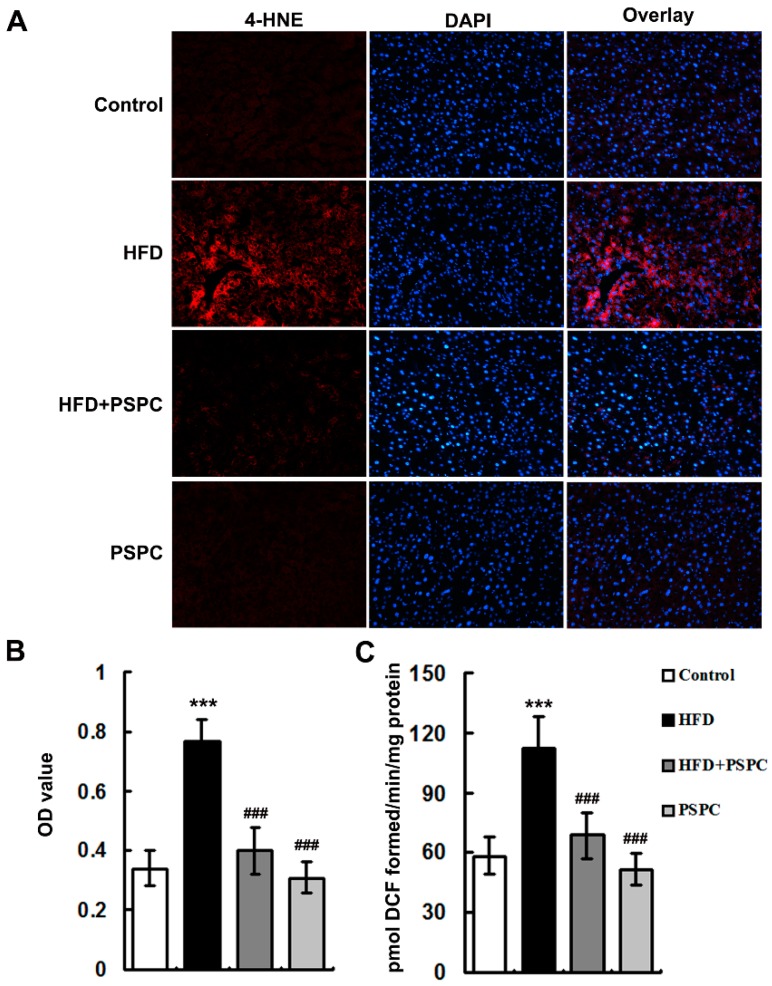
PSPC attenuates oxidative stress in HFD-treated mouse livers (*n* = 5). (**A**) 4-HNE immunofluorescence staining, 200× magnification; (**B**) 4-HNE fluorescence intensity was measured as the mean OD value; (**C**) ROS productions in mouse livers. All of the values are expressed as the mean ± SD. *** *p* < 0.001 vs. the control group; ### *p* < 0.001 vs. the HFD group.

The levels of 4-hydroxynonenal (4-HNE), a marker of lipid peroxidation, (Figure 2A) and ROS (Figure 2B) were notably elevated in the livers of HFD-treated mice, indicating the occurrence of oxidative stress. Interestingly, PSPC markedly diminished 4-HNE levels and ROS generation in the livers of HFD-treated mice (Figure 2). No significant differences in 4-HNE levels and ROS generation were found among the HFD + PSPC, PSPC and the control groups. These results indicate that PSPC remarkably attenuates oxidative stress in the livers of HFD-treated mice.

### 2.3. PSPC Restores NAD^+^ Level in HFD-Treated Mouse Livers

**Figure 3:**
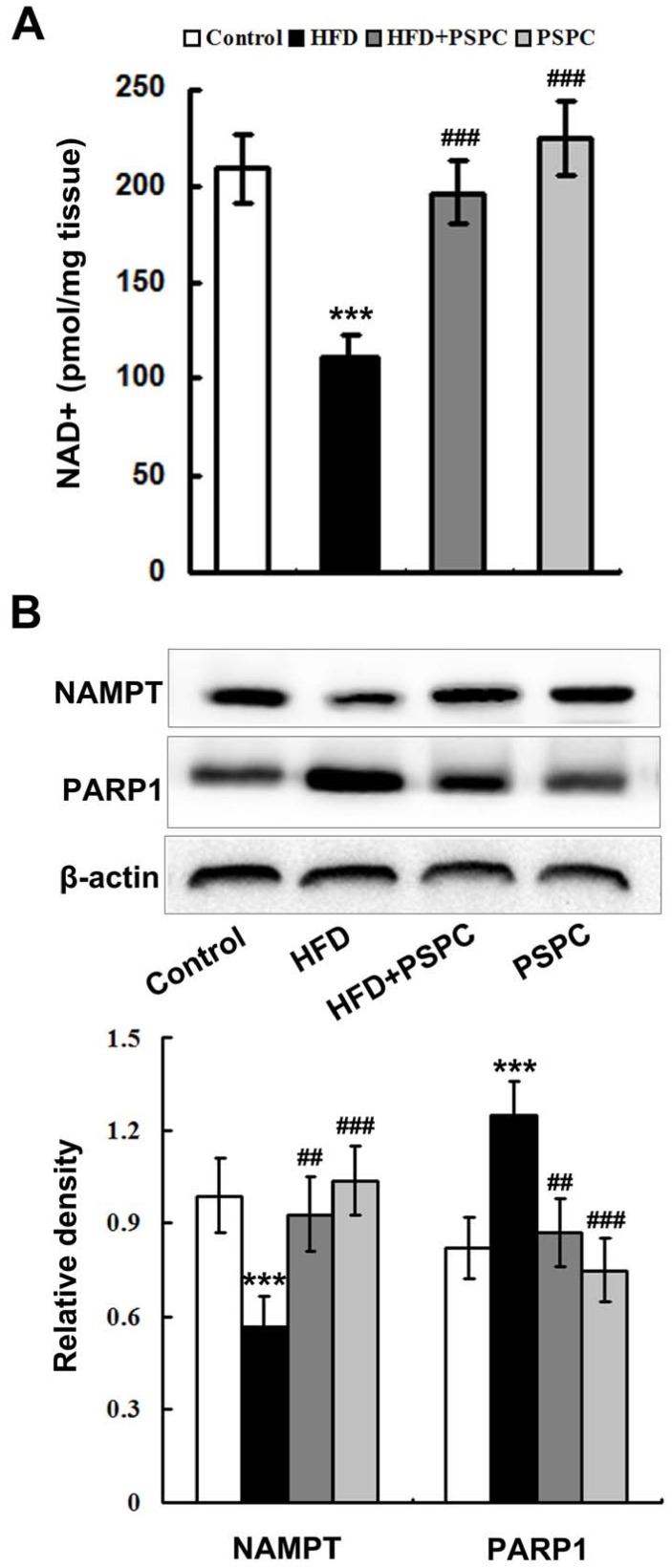
PSPC restores NAD^+^ level in HFD-treated mouse livers. (**A**) Hepatic NAD^+^ levels in all treated groups (*n* = 5); (**B**) Immunoblotting and densitometry analysis of NAMPT and PARP1 in mouse livers (*n* = 3). All of the values are expressed as the mean ± SD. *** *p* < 0.001 vs. the control group; ## *p* < 0.01, ### *p* < 0.001 vs. the HFD group.

A dramatic reduction of NAD^+^ level was found in the HFD-treated mouse liver (Figure 3A). Moreover, the protein expression of nicotinamide phosphoribosyltransferase (NAMPT), the rate-limiting enzyme of NAD^+^ salvage biosynthesis, was markedly down-regulated in the HFD-treated mouse liver (Figure 3B). Furthermore, HFD treatment notably enhanced the protein expression of poly (ADP-ribose) polymerase-1 (PARP1), the key enzyme responsible for NAD^+^ consumption, in mouse livers (Figure 3B). Interestingly, PSPC significantly restored the NAD^+^ levels and NAMPT protein expression, restrained PARP1 protein expression in the livers of HFD-treated mice (Figure 3). No significantly differences in NAD^+^ level and the protein expression of these enzymes were found among the HFD + PSPC, PSPC and the control groups. These results suggest that PSPC remarkably restored NAD^+^ levels in the livers of HFD-treated mice.

### 2.4. PSPC Depresses NAD^+^ Depletion-Mediated ER Stress in HFD-Treated Mouse Livers

**Figure 4:**
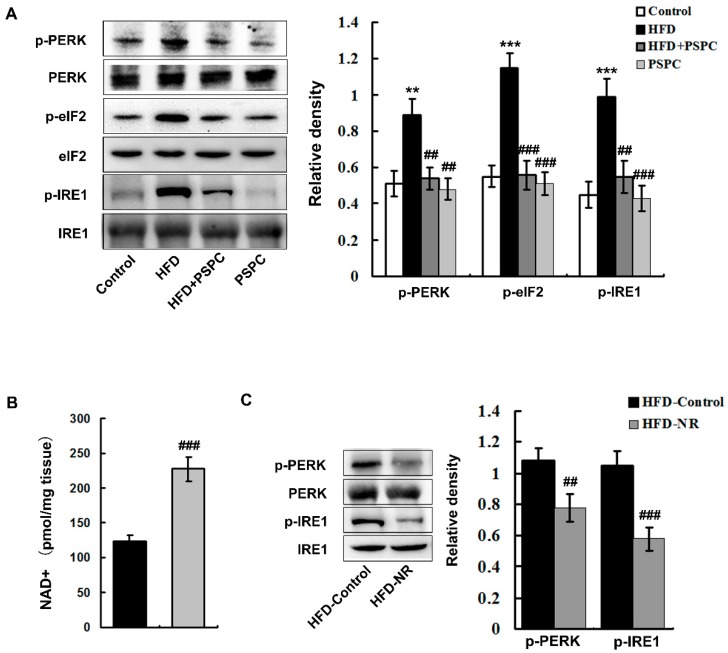
PSPC depresses NAD^+^ depletion-mediated ER stress in HFD-treated mouse livers. (**A**) Immunoblotting and densitometry analysis of ER stress markers in mouse livers (*n* = 3); (**B**) Hepatic NAD^+^ levels in all treated groups (*n* = 5); (**C**) Immunoblotting and densitometry analysis of ER stress-relative proteins in mouse livers (*n* = 3). All of the values are expressed as the mean ± SD. ** *p* < 0.01, *** *p* < 0.001 vs. the control group; ## *p* < 0.01, ### *p* < 0.001 vs. the HFD group.

HFD treatment induced a severe ER stress, which was characterized by the evidently increased protein levels of phospho-pancreatic endoplasmic reticulum resident kinase (p-PERK) (Thr980), phospho-eukaryotic translation initiation factor (p-eIF2α) (Ser51), phopho-inositol-requiring 1 (p-IRE1) (Ser724) in the HFD-fed mouse livers (Figure 4A).

To investigate whether the HFD-induced ER stress was mediated by NAD^+^ depletion, we used nicotinamide riboside (NR) to increase NAD^+^ levels in the HFD-fed mouse livers. NR treatment significantly elevated the hepatic levels of NAD^+^ in HFD-treated mice (Figure 4B). Moreover, NR treatment effectively suppressed ER stress in the livers of HFD-treated mice (Figure 4C). The above results indicated that NAD^+^ repletion abated HFD-induced ER stress in the HFD-fed mouse livers.

Interestingly, PSPC markedly decreased the protein levels of p-PERK (Thr980), p-eIF2α (Ser51), p-IRE1 (Ser724) in the HFD-fed mouse livers (Figure 4A). There were no significant differences in these ER stress markers among the HFD + PSPC, PSPC and the control groups. These results indicate that PSPC depresses NAD^+^ depletion-mediated ER stress in the livers of HFD-treated mice.

### 2.5. PSPC Abates Nuclear Factor-κB (NF-κB) p65 Nuclear Translocation and Nucleotide Oligomerization Domain Protein (NOD) Expression in HFD-Treated Mouse Livers

**Figure 5:**
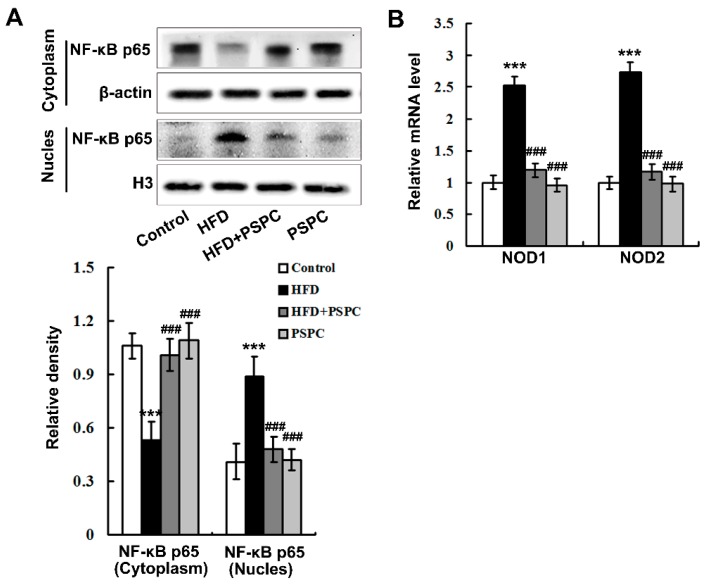
PSPC abates NF-κB p65 nuclear translocation and NOD expression in HFD-treated mouse livers (*n* = 3). (**A**) Immunoblotting and densitometry analysis of nuclear and cytoplasmic NF-κB p65 in mouse livers; (**B**) the mRNA levels of Nod1 and Nod2 in mouse livers. All of the values are expressed as the mean ± SD. *** *p* < 0.001 vs. the control group; ### *p* < 0.001 vs. the HFD group.

HFD feeding notably promoted the NF-κB nuclear translocation as indicated by the elevated nuclear and diminished cytoplasmic NF-κB p65 subunit localization in the mouse livers (Figure 5A). Furthermore, the mRNA levels of NODs, including NOD1 and NOD2, were dramatically augmented in the livers of the HFD-treated mice (Figure 5B), indicating the enhancement of NOD signaling. Interestingly, PSPC remarkably decreased NF-κB p65 nuclear translocation and NOD expression in the HFD-fed mouse livers (Figure 5). No significant differences in NF-κB p65 nuclear translocation and NOD expression were found among the HFD + PSPC, PSPC and the control groups. These results suggest that PSPC abates NF-κB p65 nuclear translocation and NOD expression in the livers of HFD-treated mice.

### 2.6. PSPC Prevents NLRP3 Inflammasome Activation in HFD-Treated Mouse Livers

**Figure 6:**
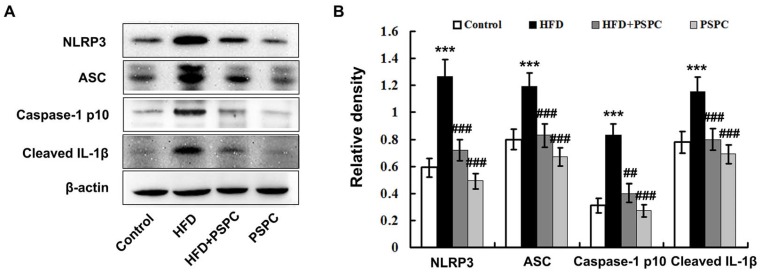
PSPC prevents NLRP3 inflammasome activation in HFD-treated mouse livers (*n* = 3). (**A**) Immunoblotting of NLRP3 inflammasome components and cleaved IL-1β in mouse livers; (**B**) Densitometry analysis of NLRP3 inflammasome components and cleaved IL-1β in mouse livers. All of the values are expressed as the mean ± SD. *** *p* < 0.001 vs. the control group; ## *p* < 0.01, ### *p* < 0.001 vs. the HFD group.

HFD treatment caused a significant increase in the active isoform of caspase-1 (10 kDA) and cleaved interleukin-1β (IL-1β) (17 kDA) levels in the HFD-fed mouse livers (Figure 6A,B). The augmentation of caspase-1 p10 by HFD was accompanied by a dramatic up-regulation of NLRP3 and ASC protein expression (Figure 6A,B), indicating that HFD markedly triggered NLRP3 inflammasome activation in the HFD-fed mouse livers. Interestingly, PSPC treatment significantly inhibited NLRP3 inflammasome activation in the HFD-fed mouse livers (Figure 6). There were no significant differences in NLRP3 inflammasome activation among the HFD + PSPC, PSPC and the control groups. These results indicate that PSPC prevents NLRP3 inflammasome activation in the livers of HFD-treated mice.

### 2.7. PSPC Suppresses Inflammation-Related Genes Expressions in HFD-Treated Mouse Livers

**Figure 7:**
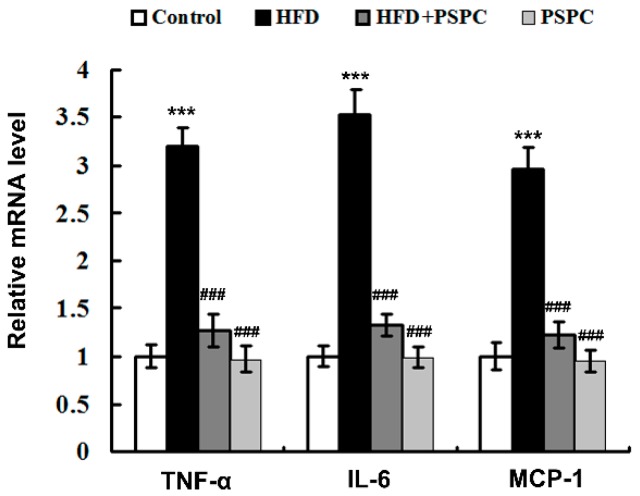
PSPC suppresses inflammation-related genes expressions in HFD-treated mouse livers (*n* = 3). The mRNA level of inflammation-related genes in mouse livers. All of the values are expressed as the mean ± SD. *** *p* < 0.001 vs. the control group; ### *p* < 0.001 vs. the HFD group.

The mRNA levels of inflammation-related genes, including tumor necrosis factor-α (TNF-α), interleukin-6 (IL-6) and monocyte chemoattractant protein-1 (MCP-1) were significantly elevated in the HFD-fed mouse livers (Figure 7). Interestingly, PSPC effectively diminished the mRNA expressions of these inflammation-related genes in the HFD-fed mouse livers (Figure 7). No significant differences in inflammation-related genes expressions were found among the HFD + PSPC, PSPC and the control groups. In combination with the abovementioned histological analysis results (Figure 1F), these results suggest that PSPC suppresses inflammatory response in the livers of HFD-treated mice.

## 3. Discussion

The importance of NLRP3 inflammasome-mediated inflammation in the development of T2D has been well demonstrated over the past few years [9,10,19]. However, the causal mechanisms underlying the activation of NLRP3 inflammasome during excess caloric consumption and obesity remain largely unknown. The present study provides novel mechanistic insights into NLRP3 inflammasome-mediated inflammation under HFD condition by revealing that NAD^+^ depletion-mediated ER stress contributed to NLRP3 inflammasome activation in the livers of HFD-treated mice. Furthermore, our findings revealed that PSPC protected against NLRP3 inflammasome-mediated inflammation via ameliorating oxidative stress-mediated NAD^+^ loss and consequent ER stress in the HFD-treated mouse livers, indicating that PSPC is a candidate for pharmacological intervention of obesity-related metabolic diseases.

There is increasing interest in the important role of NAD^+^ depletion in the pathogenesis of various injuries and diseases [5,6,7,8,20]. Recently, accumulated evidence has established that NAD^+^ depletion plays a crucial role in the development of T2D and its complications, such as steatosis and insulin resistance [5,6,7,8]. It has been well demonstrated that the protein expression of NAMPT is decreased, while the level of PARP1 is increased during HFD feeding and obesity, which leads to NAD^+^ depletion [21,22]. In the present study, our results showed that HFD markedly diminished NAMPT protein expression and augmented the level of PARP1 protein in mouse livers, which is accompanied by a decreased level of NAD^+^. Consistently, our findings suggested that HFD induced NAD^+^ depletion by abating NAD^+^ salvage biosynthesis and enhancing NAD^+^ consumption. Substantial evidences suggest that naturally occurring polyphenols exhibit beneficial effects on many aspects of T2D and its complications via strengthening NAD^+^ biosynthesis and restraining NAD^+^ breakdown pathways to boost NAD^+^ levels [23,24,25,26]. Consistent with these studies, our results revealed that PSPC dramatically restored NAD^+^ levels in the livers of HFD-treated mice by heightening NAMPT level and diminishing PARP1 level, which might be responsible for many aspects of its beneficial effects on T2D and its complications.

It is well established that intracellular NAD^+^ level is modulated by cellular stresses including oxidative stress [27,28]. Oxidative stress is reported to depress NAMPT-mediated NAD^+^ biosynthesis during metabolic syndrome and aging [29,30]. Accumulated evidence reveals that PARP1 is largely promoted by oxidative stress under various pathological conditions, resulting in NAD^+^ depletion [31,32]. In the present study, our results showed that HFD induced a severe oxidative stress, which was attenuated by PSPC in mouse livers. As strong antioxidants, naturally occurring polyphenols are well demonstrated to boost intracellular NAD^+^ levels [24,25,26]. Therefore, our findings indicated that PSPC might improve NAD^+^ metabolism to restore hepatic NAD^+^ levels in HFD-treated mice via its antioxidant effect.

ER stress is the central feature in peripheral tissues during T2D, which is responsible for many aspects of the pathogenesis of T2D and its complications. It is well established that hepatic ER stress perturbs glucose and lipid homeostasis, contributing to the development of T2D [33,34]. In this study, our results showed that HFD caused a severe ER stress in the mouse livers. Accumulated evidence indicates that naturally occurring polyphenols exhibit the strong inhibitory effects on ER stress during various pathological conditions, including T2D [18,35]. Consistently, our findings revealed that PSPC markedly attenuated HFD-induced ER stress in mouse livers, indicating that PSPC exhibited beneficial effects on T2D partly via its inhibitory effects on ER stress. Recent studies have highlighted the association between NAD^+^ loss and ER stress in nonalcoholic fatty liver disease (NAFLD), which is the hepatic manifestation of T2D [36,37]. It is suggested that NAD^+^ loss leads to the accumulation of aberrant proteins, which contributes to ER stress [38]. Moreover, the activity of Sirt1, an NAD^+^-dependent deacetylase, is compromised during NAD^+^ loss, which promotes ER stress [39]. In particular, an accumulating body of evidence demonstrates that enhancing NAD^+^ salvage biosynthesis and consequently elevating NAD^+^ level alleviate ER stress [36,37]. In the present study, the treatment of NR, a NAD^+^ precursor, largely increased the NAD^+^ levels and thereby abated ER stress in the livers of HFD-treated mice, confirming the ameliorative effects of NAD^+^ repletion on ER stress. Collectively, our findings revealed that PSPC might mitigate HFD-induced ER stress via restoring NAD^+^ levels in mouse livers.

In recent years, the important role of NLRP3 inflammasome implicated in the development of metabolic diseases including T2D received increasing attention [9,10,11,12]. NLRP3 inflammasome activation leads to autocatalytic activation of caspase-1 and the cleavage of its substrates, including IL-1β, which plays an important role in the pathogenesis of T2D and its complications. In the present study, our results revealed that HFD treatment dramatically augmented NLRP3 inflammasome activation and the expressions of inflammation-related genes in mouse livers, indicating a critical role of NLRP3 inflammasome in HFD-induced hepatic inflammation. Increasing evidence points out that ER stress activates NLRP3 inflammasome under various pathological conditions via both unfolded protein response (UPR), including IRE1 signaling, -dependent and independent pathways [40,41]. It is well established that NF-κB p65, the key transcription factor activated by ER stress, contributes to NLRP3 inflammasome activation [42]. Moreover, recent evidence indicates that ER stress provokes inflammatory response through triggering NOD1 and NOD2 signaling, which plays a role in the activation of NLRP3 inflammasome [43,44]. In this study, both NF-κB p65 nuclear translocation and NOD1/2 signaling were notably enhanced in the livers of HFD-treated mice. Our findings further suggested that HFD might induce NLRP3 inflammasome activation by promoting ER stress-mediated augment of IRE1 signaling, NF-κB p65 nuclear translocation and NOD1/2 signaling in mouse livers. It is well demonstrated that naturally occurring polyphenols effectively suppress NLRP3 inflammasome activation via their antioxidant activities [45,46]. In the present study, PSPC significantly depressed NF-κB p65 nuclear translocation, NOD1/2 signaling and NLRP3 inflammasome activation in the livers of HFD-treated mice. Thus, our findings revealed that PSPC inhibited HFD-induced NLRP3 inflammasome activation by abating ER stress-mediated augment of IRE1 signaling, NF-κB p65 nuclear translocation and NOD1/2 signaling in mouse livers.

It is well established that obesity is an important risk factor for NAFLD, and is associated with many metabolic abnormalities in liver including inflammation [47,48]. Substantial evidence suggests that obesity causes adipose tissue dysfunction, which leads to aberrant release of proinflammatory mediators, such as cytokines and specific fatty acids, contributing to the development of liver inflammation [47,49]. Moreover, obesity alters gut microbiota and increases intestinal permeability, which elevates circulating bacterial endotoxins and absorption of dietary lipids, promoting liver inflammation [50,51]. Accumulated evidence suggests that naturally occurring anthocyanins exhibit beneficial effects on metabolic abnormalities via their anti-obesity actions [52,53]. In this study, PSPC markedly decreased epididymal adipose tissue masses, as well as body weight, without influencing food intake in HFD-fed mice. Consistent with these studies, our findings indicated that PSPC might attenuate HFD-induced liver inflammation by its anti-obesity effects, which requires further study.

## 4. Materials and Methods

### 4.1. Animals and Treatment

All experimental and euthanasia procedures performed in this study were approved by the Institutional Animal Care and Use Committee of Jiangsu Normal University (Permit Number: 16-0050; 6 March 2016). ICR mice (male, 8-week-old) were purchased from Hua-fu-kang Biological Technology Co. Ltd. (Beijing, China). Mice were maintained at constant temperature (23 ± 1 °C) with a 12 h light/dark cycle and humidity (60%), and allowed access to food and drinking water ad libitum. After acclimation for one week, mice were randomly divided into four groups: Control group, HFD (60% of energy as fat; D12492; Research Diets, New Brunswick, NJ, USA) group, HFD + PSPC group, and PSPC group, and received the following treatments for 20 weeks: Mice in the Control group and the PSPC group were fed a normal diet (10% of energy as fat; D12450B; Research Diets). Mice in the HFD group and the HFD + PSPC group were fed a HFD. PSPC was purchased from Qingdao Pengyuan Natural Pigment Research Institute (Qingdao, China). The major components of PSPC by HPLC analysis are cyanidin acyl glucosides and peonidin acyl glucosides (>90%, peonidin 3-*O*-(6-*O*-(*E*)-caffeoyl-2-*O*-β-d-glucopyranosyl-β-d-glucopyranoside)-5-*O*-β-d-glucoside, peonidin 3-*O*-(2-*O*-(6-*O*-(*E*)-caffeoyl-β-d-glucopyranosyl)-6-*O*-(*E*)-caffeoyl-β-d-glucopyranoside)-5-*O*-β-d-glucopyran-oside, peonidin 3-*O*-(2-*O*-(6-*O*-(*E*)-feruloyl-β-d-glucopyranosyl)-6-*O*-(*E*)-caffeoyl-β-d-glucopyranos-ide)-5-*O*-β-d-glucopyranoside, cyanidin 3-*O*-(6-*O*-*p*-coumaroyl)-β-d-glucopyranoside) and the rest are other flavonoids), as described in our previous work [15].

#### 4.1.1. PSPC Treatment

PSPC was dissolved in in distilled water containing 0.1% Tween 80. Mice received a daily 700 mg/kg/day dose of PSPC or an equal volume of distilled water containing 0.1% Tween 80 via oral gavage (see Table 1 for details). The PSPC dosage used in this study was according to our previous work [18].

#### 4.1.2. NR Treatment

Eighteen weeks after HFD feeding, some mice of the HFD group were divided into two subgroups: HFD-control group and HFD-NR group. NR (BOC Sciences, Shirley, NY, USA) was solubilized in phosphate buffered saline (PBS). Mice received a daily 300 mg/kg/day dose of NR or an equal volume of PBS during two weeks via oral gavage (see Table 2 for details). The NR dosage used in this study was based on the literature [54,55].

Food intake was measured every three days. After 20 weeks of treatment, mice were fasted overnight, anaesthetized and sacrificed. The liver, epididymal fat and blood were immediately collected for experiments or stored at −70 °C until analysis.

### 4.2. Tissue Homogenates

The preparation of liver homogenates were performed as described in our previous work [23,56]. The protein concentration were determined using the bicinchoninic acid assay kit (Pierce Biotechnology, Rockford, IL, USA) according to the manufacturer’s instructions.

### 4.3. Biochemical Analyses

After 6 h fasting, blood samples were obtained by tail venipuncture. Fasting blood glucose concentrations were measured using an Ascensia Elite glucose meter (Bayer Corporation, Mishawaka, IN, USA). Serum insulin levels were measured with the appropriate enzyme-linked immunosorbent assay kits (ELISA; ALPCO Diagnostics, Windham, NH, USA) according to the manufacturer’s instructions. The index of the homeostasis model assessment (HOMA index) was calculated to estimate insulin resistance according to the following formula: [insulin (mIU/l) × glucose (mM)]/22.5. The serum ALT activities were spectrophotometrically measured with a diagnostic kit (Jiancheng Institute of Biotechnology, Nanjing, China) following the manufacturer’s instructions.

### 4.4. ROS Assay

The amounts of ROS were quantified as previously described based on the oxidation of 2′,7′-dichlorodihydrofluorescein diacetate (H_2_-DCF-DA) to 2′,7′-dichlorofluorescein (DCF) [23,56]. Briefly, the homogenate was diluted 1:20 (*v*/*v*) with ice-cold Locke’s buffer [154 mM NaCl, 5.6 mM KCl, 3.6 mM NaHCO_3_, 2.0 mM CaCl_2_, 10 mM D-glucose and 5 mM 4-(2-hydroxyethyl)-1-piperazine-ethanesulfonic acid, pH 7.4] to obtain a tissue concentration of 10 mg/mL. The reaction mixture (1 mL) containing Locke’s buffer, 0.2 mL of homogenate and 10 μL of 5 mM H_2_-DCF-DA was incubated for 15 min at room temperature to allow the H_2_-DCF-DA to be incorporated into any membrane-bound vesicles and the diacetate group to be cleaved by esterases. After 30 min of further incubation, the conversion of H2-DCF-DA to the fluorescent product DCF was measured using a Molecular Devices M2 plate reader (Molecular Devices Corporation, Menlo Park, CA, USA) with excitation at 484nm and emission at 530 nm. Background fluorescence (conversion of H_2_-DCF-DA in the absence of homogenate) was corrected by the inclusion of parallel blanks. ROS formation was quantified from a DCF standard curve. ROS level was expressed as pmol DCF formed/min/mg protein.

### 4.5. Liver Slice Collection and Histopathological Analysis

Liver slice collection and hematoxylin-eosin staining were performed according to the protocols described in our previous work [23,56]. The liver sections stained with hematoxylin-eosin (Sigma-Aldrich, St. Louis, MO, USA) were examined by an expert liver pathologist blinded to the treatment groups.

### 4.6. Immunofluorescence Staining

The preparation of frozen sections and immunofluorescence staining were performed as described previously [23,56]. The liver sections were incubated with the primary antibody (rabbit anti-4-HNE antibody, 1:100, Alpha Diagnostics, San Antonio, TX, USA) overnight at 4 °C. After a washing with phosphate buffered saline, the liver sections were incubated with Texas Red-conjugated anti-rabbit IgG (1:200, Vector Laboratories, Inc., Burlingame, CA, USA).

### 4.7. NAD^+^ Assay

NAD^+^ levels were measured using EnzyChromTM NAD^+^/NADH Assay kit (BioAssay Systems, Hayward, CA, USA) following the manufacturer’s instructions. Briefly, ~20 mg liver tissue from each sample was homogenized in 100 μL NAD extraction buffer. Liver extracts were heated at 60 °C for 5 min and were then neutralized by the addition of 100 μL of NADH and 20 μL assay buffer. The NAD containing supernatants were obtained by centrifuging the liver extracts at 14,000 rpm for 5 min. 80 μL working reagent was added to 40 μL of NAD standard and samples in a 96-well plate. OD at time “zero” was read at 565 nm. OD15 was read after a 15-min incubation at room temperature. The OD0 values were subtracted from OD15 values for concentration analysis. The NAD levels of unknown samples were calculated from the standard curve and expressed as pmol/mg liver.

### 4.8. Quantitative Real Time Polymerase Chain Reaction

The quantitative real time polymerase chain reaction was performed as described previously [23,56]. The primers used were: NOD1, Forward: 5′-TGACGTTCCTGGGTTTATACAACA-3′, Reverse: 5′-CCAGGATTTGGGCCACATAC-3′; NOD2, Forward: 5′-CCTGGTACGTGCCCAAAGT AG-3′, Reverse: 5′-GCCAAGTAGAAAGCGGCAAA-3′; TNF-α, Forward: 5′-TCTCATTCCTGCTTGT GG-3′, Reverse: 5′-ACTTGGTGG TTT GCTACG-3′; IL-6, Forward: 5′-CCAGAGATACAAAGAAAT GATGG-3′, Reverse: 5′- ACTCCAGAAGACCAGAGGAAAT -3′; MCP-1, Forward: 5′-AGGTCCCT GTCATGCTTCTG-3′, Reverse: 5′-GCTGCTGGTGATCCTCTTGT-3′; β-actin, Forward: 5′-TGCTGTC CCTGTATGCCTCTG-3′, Reverse: 5′-TTGATGTCACGCACGATTTCC-3′. The relative levels of target mRNAs, were normalized to β-actin mRNA, and were calculated by the comparative cycle threshold (Ct) method.

### 4.9. Western Blot Analysis

The western blot analyses were performed as described in our previous work [23,56]. Briefly, samples (50 μg protein) were separated on denaturing SDS-PAGE gels and transferred to polyvinylidene difluoride (PVDF) membranes (Roche Diagnostics Corporation, Indianapolis, IN, USA) by electrophoretic transfer. The membrane was blocked with 5% non-fat milk or bovine serum albumin (BSA) in 0.1% Tween-20/TBS followed by overnight incubation with primary antibodies: rabbit anti-NAMPT, rabbit anti-PARP1, rabbit anti-ASC, rabbit anti-caspase-1 p10, and goat anti-cleaved IL-1β antibodies (Santa Cruz Biotechnology, Santa Cruz, CA, USA); rabbit anti-p-PERK (Thr980), rabbit anti-total-PERK, rabbit anti-total-eIF2α, rabbit anti-NF-κB p65, rabbit anti-histone H3 and rabbit anti-β-Actin antibodies (Cell Signaling Technology, Beverly, MA, USA); rabbit anti-p-eIF2α (Ser51), rabbit anti-p-IRE1 (Ser724) and rabbit anti-total-IRE1 antibodies (Abcam, Cambridge, UK); rabbit anti-NLRP3 antibodies (Novus, Littleton, CO, USA). After washing, proteins were detected using HRP-conjugated anti-rabbit (Cell Signaling Technology), and HRP-conjugated anti-goat (Santa Cruz Biotechnology) secondary antibodies. Immunoreactive proteins were visualized using 20 × LumiGLO^®^ Reagent and 20 × Peroxide (Cell Signaling Technology). The optical density (OD) values of the detected bands were measured with Scion Image analysis software (Scion Corp., Frederick, MD, USA). The OD values were normalized using appropriate internal controls (optical density detected protein/optical density internal control).

### 4.10. Statistical Analysis

All statistical analysis was performed using SPSS version 11.5 (SPSS Inc., Chicago, IL, USA). All the data were analyzed with a one-way ANOVA followed by Tukey’s Honestly Significant Difference (HSD) post-hoc test and Student’s *t*-test. Data were expressed as means ± standard deviation (SD). Statistical significance was set at *p* < 0.05.

## 5. Conclusions

In summary, our results revealed that PSPC exhibits significant beneficial effects on HFD-induced hepatic inflammation by diminishing oxidative stress, consequently boosting NAD^+^ level to abate ER stress-mediated augment of IRE1 signaling, NF-κB p65 nuclear translocation and NOD1/2 signaling, ultimately lowering NLRP3 inflammasome activation and consequent expressions of inflammation-related genes. This study provides novel mechanistic insights into pathogenesis of HFD-induced inflammation and indicates that PSPC is a candidate for pharmacological intervention of obesity-related metabolic diseases.

## Figures and Tables

**Table 1:** Details of PSPC treatment.

	Diet		Treatment (Gavage)		
Group	HFD	ND	PSPC (700 mg/kg/day)	Distilled Water Containing 0.1% Tween 80	Exposure Time
Control		**+**		**+**	0–20 weeks
HFD	**+**			**+**	0–20 weeks
HFD + PSPC	**+**		**+**		0–20 weeks
PSPC		**+**	**+**		0–20 weeks

**Table 2:** Details of NR treatment.

	Diet		Treatment (Gavage)		
Group	HFD	ND	NR (300 mg/kg/day)	PBS	Exposure Time
HFD-Control	**+**			**+**	19–20 weeks
HFD-NR	**+**		**+**		19–20 weeks
